# Diuretics, Ca-Antagonists, and Angiotensin-Converting Enzyme Inhibitors Affect Zinc Status in Hypertensive Patients on Monotherapy: A Randomized Trial

**DOI:** 10.3390/nu10091284

**Published:** 2018-09-11

**Authors:** Joanna Suliburska, Katarzyna Skrypnik, Monika Szulińska, Justyna Kupsz, Leszek Markuszewski, Paweł Bogdański

**Affiliations:** 1 Institute of Human Nutrition and Dietetics, Poznan University of Life Sciences, Wojska Polskiego St. 31, 60-624 Poznań, Poland; katarzyna.skrypnik@gmail.com; 2 Department of Treatment of Obesity, Metabolic Disorders and Clinical Dietetics, Poznan University of Medical Sciences, Szamarzewskiego St. 82/84, 60-569 Poznań, Poland; mszulinska1@wp.pl (M.S.); pawelbogdanski73@gmail.com (P.B.); 3 Department of Physiology, Poznan University of Medical Sciences, Święcickiego St. 6, 61-781 Poznań, Poland; justynakup@o2.pl; 4 Department of Endocrinology and Metabolic Diseases, Polish Mother’s Memorial Hospital—Research Institute, 281/289 Rzgowska St., 93-338 Łódź, Poland; leszekmarkuszewski@gmail.com

**Keywords:** antihypertensive pharmacotherapy, mineral metabolism, zinc

## Abstract

Background: Antihypertensive drugs affect mineral metabolism, inflammation, and the oxidative state. The aim of this study was to evaluate the effects of antihypertensive monopharmacotherapy with diuretics, β-blockers, calcium antagonists (Ca-antagonists), angiotensin-converting enzyme inhibitors (ACE-I), and angiotensin II receptor antagonists (ARBs) on zinc (Zn), iron (Fe), and copper (Cu) status, parameters of oxidative and inflammatory states, and glucose and lipid metabolism in patients with newly diagnosed primary arterial hypertension (AH). Methods: Ninety-eight hypertensive subjects received diuretics, β-blockers, Ca-antagonists, ACE-I, or ARB for three months. Zn, Fe, and Cu concentrations were determined in blood, urine, and hair. Results: A decrease in zinc serum and erythrocyte concentration and an increase in zinc urine concentration were registered after diuretic administration. Ca-antagonists led to a decrease in erythrocyte zinc concentration. A decrease in serum zinc concentration was observed after ACE-I. A decrease in triglyceride serum concentration was noted after ACE-I therapy, and a decrease in tumor necrosis factor-α serum concentration was seen following administration of Ca-antagonists. Hypotensive drugs led to decreases in catalase and superoxide dismutase serum concentrations. Conclusions: Three-months of monotherapy with diuretics, Ca-antagonists, or ACE-I impairs zinc status in patients with newly diagnosed primary AH. Antihypertensive monopharmacotherapy and zinc metabolism alterations affect lipid metabolism, the oxidative state, and the inflammatory state.

## 1. Introduction

Antihypertensive drugs affect the mineral metabolism of iron (Fe); copper (Cu); and, especially, zinc (Zn) [1,2,3,4,5,6,7,8,9,10]. Diuretics increase Zn excretion in the urine [1,10] and decrease Zn serum concentrations [2,10]. Atenolol decreases serum Zn levels [3,10]. Neither atenolol nor metoprolol have an effect on erythrocyte Zn content [3,4,10]. Captopril increases renal Zn loss [6,10] and decreases Zn concentration in erythrocytes [6,10] and (similarly to ramipril [4,10]) in plasma [5,10]. Decreased intracellular Zn levels [7,10] and increased renal Zn excretion were also observed after enalapril administration [6,10]. Perindopril normalizes increased zincuria [8,10]. An increase in Zn loss in the urine after losartan use [9,10] and reduced Zn plasma and erythrocyte concentrations after valsartan use [4,10] have been reported. Amlodipine has no effect on plasma or erythrocyte Zn measurements [4,10]. Verapamil has no effect on erythrocyte Zn, but a decrease in serum Zn concentration has been observed after therapy with this drug [3,10]. The cited studies were performed without dietary control, which makes it impossible to determine the effect of Zn intake on the results. In our previous studies on patients with arterial hypertension (AH) undergoing pharmacotherapy, we observed decreased Zn levels in the serum [11] and hair [12,13], as well as increased Zn excretion in the urine [11,13]. However, to date, no well-planned human studies have been performed on patients with newly diagnosed primary AH, who were not encumbered with other medical states, with the aim of determining the effects of monotherapy with particular hypotensive drug groups on zinc, iron, or copper mineral status [11,12,13]. 

Antihypertensive treatment modulates the antioxidant status and the inflammatory state. Diuretics, β-blockers, angiotensin-converting enzyme inhibitors (ACE-I), and calcium antagonists (Ca-antagonists) act to increase the activity of superoxide dismutase (SOD) and catalase (CAT) [14]. The inhibition of angiotensin II receptors increases the activity of enzymes with antioxidant properties [15]. Unbalanced Zn and Cu metabolism negatively affects the activity of CAT, SOD, and carbonic anhydrase (CA) [16], and, in human serum, SOD activity is a marker of Zn status [17]. In hypertensive patients, Ca-antagonists show an anti-inflammatory action [17,18]. In people being treated for AH, mineral balance has a significant effect on glucose and lipid metabolism [12]. A negative correlation between Cu serum level and serum glucose concentration has been observed [11]. Hypertensive patients undergoing antihypertensive pharmacotherapy show a positive correlation between serum high-density lipoprotein (HDL) concentration and serum and hair Zn content, a negative correlation between serum HDL concentration and hair Fe content, and a positive correlation between serum triglyceride (TG) concentration and hair Cu content [12]. However, the effects of particular antihypertensive drug groups on the connections between mineral, lipid, and glucose metabolism are frequently ignored and remain uninvestigated.

The aim of our study was to evaluate the effect of three-months of antihypertensive monopharmacotherapy with diuretics, β-blockers, Ca-antagonists, ACE-I, and angiotensin-II receptor antagonists (ARB) on zinc, iron, and copper status, as well as on selected biochemical parameters of oxidative and inflammatory states and of glucose and lipid metabolism in patients suffering from newly diagnosed primary AH with no previous hypotensive therapy. To the best of our knowledge, this study is the first human trial anywhere to investigate the effect of particular antihypertensive drug groups on mineral status in such a homogenous group of patients.

## 2. Materials and Methods 

### 2.1. Study Patients

The study was approved by the Ethics Committee of Poznań University of Medical Sciences (approval no. 86/09) and complied with the ethical guidelines described in the Declaration of Helsinki and its amendments. The study has been registered on ClinicalTrials.gov under the ID NCT03465462. The study protocol can be accessed at https://clinicaltrials.gov/ct2/show/NCT03465462.

Patients who had not previously undergone antihypertensive therapy (*n* = 425) were screened at the outpatient clinic of the Department of Internal Medicine, Metabolic Disorders, and Hypertension at Poznań University of Medical Sciences between January and September of 2016. A total of 105 patients were enrolled in the study. The inclusion criteria were informed written consent, primary arterial hypertension diagnosed in accordance with the 2013 guidelines of the European Society of Hypertension [19], no previous antihypertensive therapy, 18–65 years of age, and stable body weight (<3 kg change in self-reported weight in the three months prior to enrollment). The exclusion criteria were a diagnosis of secondary arterial hypertension; the use of mineral supplements in the three months prior to enrollment; treatment of lipid disorders in the three months prior to the study; a history of stroke, ischemic heart disease, congestive heart failure, peripheral artery or vein disease, clinically significant conduction disorders, arrhythmia, or diabetes mellitus; abnormal function of the liver, kidneys, or thyroid gland; clinically significant acute or chronic inflammation within the digestive, respiratory, genitourinary tract, or in the oral cavity, pharynx, larynx, or paranasal sinuses; arthritis, connective tissue diseases, or malignancy; any known infection in the month prior to enrollment; a history of pacemaker implantation; nicotine, drug, or alcohol abuse; pregnancy, parturition, or lactation at enrollment or in the three months prior to enrollment; mental disorders; or any other state that, in the opinion of the investigators, would make participation in the study not in the best interest of the subject, or could prevent, limit, or confound the efficacy of the study. The occurrence of any of the exclusion criteria during the study resulted in immediate withdrawal of the subject from the trial.

### 2.2. Study Design

This two-stage parallel study was carried out as a prospective randomized trial. In the first stage, primary arterial hypertension was diagnosed. In the second stage, which lasted three months, patients underwent antihypertensive monotherapy. Drugs from five antihypertensive groups were used: diuretics, ACE-I, ARB, Ca-antagonists, and β-blockers. The numbers of patients given each type of drug are shown in Table 1. The primary outcomes of the study were Fe, Zn, and Cu content of serum, erythrocyte, hair, and urine. The study’s secondary outcomes were body mass, height, waist and hip circumference, blood pressure, selected serum biochemical parameters: total cholesterol (TCH), low-density lipoprotein cholesterol (LDL), HDL, TG, glucose (GLU), albumin (Alb), C-reactive protein (CRP), tumor necrosis factor α (TNF-α), ferritin (Ferr), ceruloplasmin (Cer), total iron binding capacity (TIBC), nitric oxide (NO), CA, CAT, SOD, white blood cell count (WBC), red blood cell count (RBC), hemoglobin (Hgb), and hematocrit (HCT). 

Subjects were monitored by a dietician and were asked to not alter their diet. Moreover, subjects were instructed not to take dietary supplements and to maintain their current level of physical activity and lifestyle. On the last day of both stages of the trial, blood, hair, and urine samples were collected from the patients, and the BP was measured. Three days prior to collection of the blood samples, in the first and second stages of the trial, a 24-h dietary recall was obtained from the patients to determine dietary intake. The dietary recall system developed by the Polish National Food and Nutrition Institute was used. A standard album of meal portion sizes was applied. In the first stage, dietary recall was retrospective; in the second stage, a food diary was used. Nutrient contents were determined by a dietician using a computer program (Dietetyk 3.0, Alpha-Net Software, Gdansk, Poland). In order to increase patient compliance, all packages of hypotensive drugs were retrieved on the last day of the second stage of the study and the tablets counted. Furthermore, patients were instructed to note the time and hour of drug administration in a special drug diary. The compliance rate of all patients was 80–100%. No changes were made to the methods once the trial had commenced.

### 2.3. Anthropometry

In the first stage of the study, subjects’ body mass, height, waist circumference, and hip circumference were measured in the morning, after night-long rest, with the last meal having been consumed twelve hours before. During the measurement, patients were unshod and wore light clothes. Height was determined to the nearest 1 cm, and weight was determined to the nearest 0.1 kg. BMI was calculated by dividing the weight (kg) by the height (m) squared. Waist circumference (cm) was measured at the midpoint between the lower margin of the last palpable rib and the top of the iliac crest at the end of normal expiration. Hip circumference was measured around the widest part of the buttocks. Waist and hip circumferences were measured to the nearest 0.5 cm. WHR was calculated as the waist circumference divided by the hip circumference [20]. 

### 2.4. Blood Pressure Measurement

Blood pressure was measured using a digital electronic tensiometer (705IT, Omron, Kyoto, Japan) with large or regular cuffs, depending on the subject’s arm circumference. The guidelines of the European Society of Hypertension were used [19]. Blood pressure measurements were performed after fifteen minutes’ rest in the morning after nightlong rest and fasting. During the measurement, patients took a sitting position with the back and arm supported and lower limbs uncrossed. Blood pressure was taken as the mean of three subsequent measurements. 

Twenty-four-hour ambulatory blood pressure monitoring (ABPM) was performed in all patients only in the first stage of the study, due to the confirmed diagnoses of hypertension. 

### 2.5. Collection of Blood Samples

Blood samples were taken in the morning, after 30 min in a supine position following nightlong rest and twelve hours of fasting. The blood was collected from an ulnar vein into serum-separated tubes, and into heparin sodium tubes to obtain erythrocytes. The coagulated blood was left to clot at room temperature and then centrifuged. The supernatant fluid was separated. Serum samples were frozen and stored for analysis at −20 °C.

To separate the erythrocytes, the total blood was centrifuged for 15 min at 4 °C at 2000× *g*. Blood cells were washed three times with 5 mL of 0.9% saline solution and centrifuged for 10 min at 2000× *g* at 4 °C. After centrifuging, the saline solution was removed, and the erythrocyte mass was placed in demineralized Eppendorf tubes. The separated erythrocytes were frozen at −20 °C and stored for mineral analysis. The blood sample collection procedure was described in our previous paper [11].

### 2.6. Collection of Urine Samples 

On the last day of each stage of the study, a 24-h urine collection was performed. Urine samples were collected after a night’s rest and 12-h fast into sterilized tubes and stored at 4 °C. The volume of 24-h urine collection was registered. A representative sample of 24-h urine collection was separated and stored at −20 °C for further analysis. The entire urine collection procedure was described in our previous paper [11].

### 2.7. Collection of Hair Samples 

Before hair samples were collected, subjects were asked to wash their hair using a shampoo containing no functional components, according to a standardized washing procedure. It was explained to them that reliable results could only be obtained by complying with this procedure. The use of hairspray, perms, and hair dye was forbidden during the study. A hair strand of 1 cm length, measuring from the occiput scalp, was collected and placed into a labeled paper bag. Hair samples were washed in acetone and deionized water, dried at 105–110 °C, and weighed. 

### 2.8. Mineral Determination

Prior to determining the iron, zinc, and copper levels, the samples of serum, urine, erythrocytes, and hair were digested in 65% (*w*/*w*) spectra pure HNO_3_ (Merc, Kenilworth, NJ, USA) using a Microwave Digestion system (MARS 5, CEM, Matthews, NC, USA). After digestion, flame atomic absorption spectrometry (AAS-3, Carl Zeiss, Jena, Germany) was used to determine the concentrations of iron, zinc, and copper in the mineral solutions. The mineral contents of serum and tissues were measured at the following wavelengths: 248.3 nm for iron, 213.9 nm for zinc, and 324.8 nm for copper. The accuracy of the method was verified with certified reference materials (HUM ASY CONTROL 2 and URN ASY CONTROL 2, Seronorm Trace Elements Whole Blood L-2, Sero, Billingstad, Norway) and proved to be 95–98% for iron, 95–96% for zinc, and 99–103% for copper. The procedure for determining mineral content was presented in our previous paper [11].

### 2.9. Biochemical Measurements

The serum concentrations of TCH, HDL, and TG were measured using routine enzymatic methods. The measurements were performed using commercial kits (Abcam, Cambridge, UK). TCH serum level was estimated by the reaction with cholesterol esterase, cholesterol oxidase, and peroxidase. HDL serum concentration was estimated by the direct enzyme method. TG serum concentration was measured using the enzymatic method with lipase, glycerol kinase, phospho-glycerol-3-oxidase, and peroxidase. The serum concentration of LDL was calculated using the Friedewald formula: LDL (mmol/L) = TCH (mmol/L) − HDL (mmol/L) − TG (mmol/L)/2.2 [21]. GLU serum concentration was estimated using the radioimmunological method with commercial kits (Abcam, Cambridge, United Kingdom).

Erythrocyte CAT was determined using a spectrophotometric method to measure the breakdown of hydrogen peroxide by a catalase; a commercial kit was employed (Oxis International, CA, USA). Erythrocyte enzyme activity was determined as units per gram of Hgb. SOD activity was determined in the erythrocyte hemolysate using a modification of the epinephrine–adenochrome detection system (Oxis International, CA, USA). CRP serum concentration was measured using a turbidimetric immunoassay commercial kit (R&D System, Minneapolis, MN, USA). Serum NO concentration was measured with the aid of an enzyme immunoassay commercial kit (R&D Systems, Minneapolis, MN, USA). CA, TNF-α, and albumin serum concentrations were determined by enzyme-linked immunosorbent assay (ELISA) using commercial kits (R&D System, Minneapolis, MN, USA). Serum ceruloplasmin and ferritin concentrations were measured using the ELISA method with commercial kits (Abcam, Cambridge, United Kingdom). TIBC in serum was determined by the ELISA method with a commercial kit (MyBioSource, San Diego, CA, USA). Spectrometric measurements were performed using an Advia 1800 device (Siemens, Berlin, Germany).

Hemoglobin concentration (mg/mL) was measured using the cyanmethemoglobin method (Merck, Darmstadt, Germany). A complete blood count was performed using a hematology analyzer (Cell-Dyn 3700, Abbott Laboratories, Lake Bluff, IL, USA). 

The accuracy and precision of the techniques used to assay the biochemical parameters were validated. Reproducibility was checked against a human serum control (Randox Laboratories, Crumlin, UK). Accuracy was assessed by means of the recovery value, which ranged between 95% and 109%. The variability coefficient did not exceed 10%. The biochemical analysis procedure was described in our previous paper [11].

### 2.10. Statistical Analysis

Statistical for Windows 10.0 (StatSoft, Kraków, Poland) was used to carry out the statistical analysis. The results are presented here as arithmetic means ± standard deviations (SDs). The Shapiro–Wilk test was used to check the normal distribution of the data. Comparisons between groups were carried out using the unpaired *t*-test (for data with normal distribution) or the Mann–Whitney *U*-test. The paired *t*-test (for data with normal distribution) or the Wilcoxon rank–sum test was used to determine the statistical significance of the variables between the first and the second stages of the study. A *p*-value of less than 0.05 was regarded as significant, and the α level for the tests was 0.05. Before enrollment, the statistical power of the test used in the study was calculated, which allowed for a sufficient number of patients to be enrolled to each group. The size (*n*) of each study group is presented in Table 1. The analysis was performed for the originally assigned groups. There were no changes to the trial outcomes after the trial commenced.

### 2.11. Randomization

Patients were allocated to this open-label study intervention by physicians specializing in hypertensiology, who enrolled participants and who assigned participants to interventions. Patients received the study drugs according to medical recommendations and in line with the 2013 European Society of Hypertension guidelines [19]. Patients’ medical indications and contraindications were considered to match the individual patients to the most medically appropriate study drug with the highest safety and hypotensive effectiveness. Thus, medical and ethical conscientiousness, compliance with current therapy standards, high quality of treatment, and patients’ medical safety were the only criteria taken into consideration during the randomization process. Apart from the differences in the drugs used, the interventions did not differ.

## 3. Results

**Figure 1:**
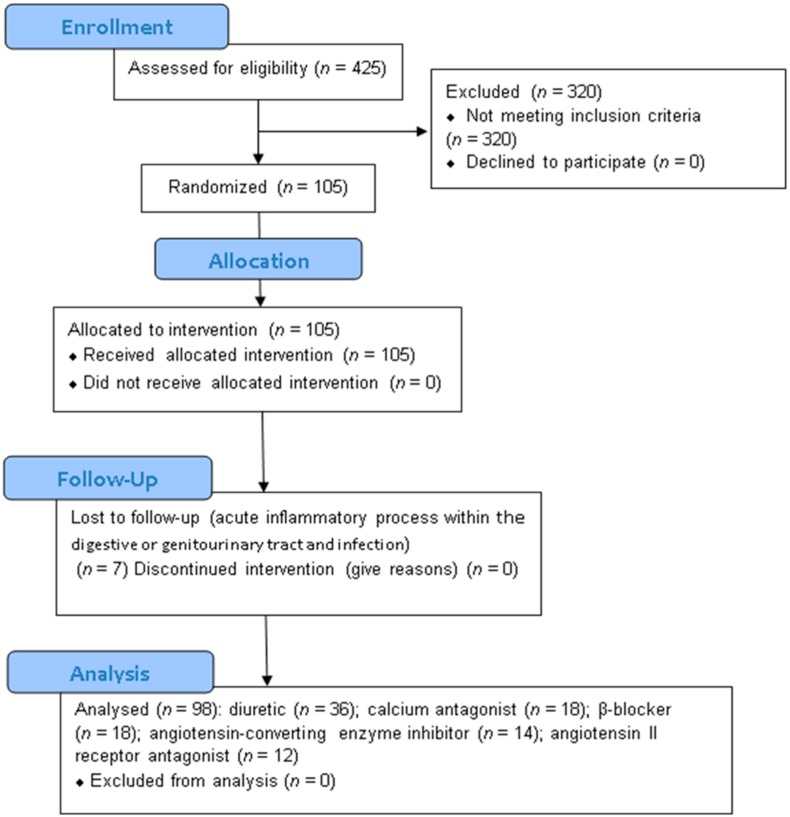
Flow diagram of the study.

Four hundred and twenty-five patients were examined at the outpatient clinic of the Department of Internal Medicine, Metabolic Disorders and Hypertension, Poznań University of Medical Sciences. The recruitment period lasted from January to September 2016. A total of 320 individuals were excluded from the trial due to their use of mineral supplements within the three months prior to enrollment (22); a secondary form of hypertension (5); lipid disorders requiring treatment in the three months prior to the trial (15); a history of ischemic heart disease (55), congestive heart failure (21), stroke (18), clinically significant conduction disorders, or arrhythmia (19); having a pacemaker (14), peripheral artery or vein disease (9), diabetes mellitus (44); abnormal renal (8), liver (7), or thyroid gland (11) function; clinically significant chronic or acute inflammatory process within the respiratory (2), genitourinary (3), or digestive tract (3), or in the oral cavity, larynx, pharynx, or in the paranasal sinuses (9); connective tissue diseases (3), arthritis (2), or malignancy (1); nicotine, alcohol, or drug abuse (19); infection in the month prior to enrollment (26); pregnancy, childbirth, or lactation at enrollment or in the three months prior to enrollment (2); and mental disorders (2). One hundred and five subjects met all the inclusion criteria and none of the exclusion criterion, were enrolled into the study, and received the study intervention. After enrolment, seven subjects were excluded from the trial due to acute inflammatory process within the digestive (3) or genitourinary (2) tract or infection (2). Ninety-eight subjects (61 women and 37 men) completed the study and underwent statistical analysis. The baseline characteristics of the study population and the number of patients receiving monotherapy from particular antihypertensive drug groups are presented in Table 1. The follow-up period lasted from January to December of 2016. The trial ended when the intervention of the last subject was complete. There were no significant differences in age, body mass index (BMI), or waist–hip ratio (WHR) between patients receiving monotherapy with particular antihypertensive drug groups. No significant differences were registered in the intake of nutrients, energy, Fe, Zn, and Cu between patients receiving monotherapy from particular antihypertensive drug groups in the first or second stages of the trial, or between stages of the trial. No significant harms or unintended effects were registered in any of the groups. A flow diagram of the study is presented in Figure 1.

### 3.1. Blood Pressure Measurements

All the antihypertensive drug types caused significant decreases in both systolic and diastolic blood pressure (BP) after the second stage of study. Blood pressure measurements are presented in Table 2. There were no significant differences in blood pressure between patients receiving monotherapy from particular antihypertensive drug groups, in either stage of the study.

### 3.2. Mineral Measurements

In the first stage of the study, there were no significant differences in Fe, Zn, or Cu content in serum, erythrocytes, urine, or hair between patients receiving monotherapy from the different antihypertensive drug groups. In the second stage of the study, when particular drug groups were examined, a significant decrease was registered in zinc concentration in serum and erythrocytes, while a significant increase was seen in zinc urine concentration in patients treated with a diuretic. Ca-antagonists led to a significant decrease in erythrocyte zinc concentration. A significant decrease in serum zinc concentration was observed after monotherapy with ACE-I. The results of Fe, Zn, and Cu measurements are presented in Table 3.

### 3.3. Biochemical Measurements

In the first stage, there were no significant differences in either biochemical or complete blood count parameters between patients receiving monotherapy from the different antihypertensive drug groups. In the second stage of the study, a decrease was registered in TG serum concentration after ACE-I therapy and in TNF-α serum concentration after Ca-antagonist therapy. Moreover, Ca-antagonists led to an increase in white blood cell count, though this did not exceed the leukocytosis level. All five groups of studied hypotensive drugs led to a significant decrease in CAT and SOD serum concentrations, except that there was no change in CAT serum concentration after treatment with ARB. Diuretics, β-blockers, ACE-I, and ARB led to significant increases in serum NO concentration. Details of the biochemical and complete blood count parameters are presented in Table 2.

## 4. Discussion

Our study is the first to examine the effects of particular groups of antihypertensive drugs on Zn, Fe, and Cu content in serum, erythrocytes, urine, and hair in a single trial involving human hypertensive subjects who had not previously undergone hypotensive treatment. The main findings of the study are that there were alterations of Zn in serum, erythrocytes, and urine after treatment with diuretics, ACE-I, and Ca-antagonists. 

Zinc metabolism in humans is involved in the regulation of BP and the development of AH [22,23]. BP inversely correlates with serum zinc concentration, and so Zn exerts an antihypertensive effect [24]. On the other hand, increased zinc serum concentration has been reported in elderly people with isolated systolic arterial hypertension [25].

The interactions between antihypertensive drugs and Zn metabolism have a pharmacokinetic basis [10]. In our trial, we documented an increase in urine Zn loss and a decrease in serum and erythrocyte Zn concentration following pharmacotherapy with diuretics. Thiazides are among the most frequently used diuretics globally, and the majority of subjects in our trial undergo treatment with diuretics used indapamide, a thiazide-like diuretic. Thiazides inhibit NaCl transport in the distal tubule, where Zn reabsorption is also disturbed, which results in a significant increase in zincuria [10]. Elevated zincuria may be responsible for the decreased serum Zn levels observed following use of hydrochlorothiazide [2]. Taking into consideration the fact that hypertensive patients have abnormal Zn homeostasis with increased Zn excretion in the urine [8], we can hypothesize that thiazides and thiazide-like diuretics may intensify Zn deficiency in AH, despite the normalization of BP. The decrease in erythrocyte Zn concentration in hypertensive patients treated with diuretics is clearly a new finding of our study and seems to be an expression of a pronounced Zn deficit.

In our trial, decreased serum Zn concentrations were registered following the use of ACE-I. The different alterations in Zn metabolism caused by various ACE-Is are the results of structural differences between drugs from this group. ACE-Is are structurally divided into sulfhydryl-containing ACE-Is (captopril), phosphonate-containing ACE-Is (fosinopril), and dicarboxyl-containing ACE-Is (perindopril, ramipril, and enalapril) [10]. It has been proposed that the sulfhydryl group is a chelating agent that binds to bivalent metals, here Zn, resulting in elevated urinary Zn loss. This theoretical model explains why captopril has a greater effect on serum, urine, and erythrocyte Zn than enalapril [7]. In light of these facts, the predominance in our trial of treatment with perindopril and ramipril over captopril seems to be the reason for the lack of change in the urine and erythrocyte Zn levels in the ACE-I group. One study reported intensified zincuria following losartan use, which seems to show that the sulfhydryl group is not necessary to increase the excretion of Zn in the urine [9]. In our trial, however, we found no changes in urinary Zn levels in patients using ARB. We suggest that the lower activity of Na/H antiporter in the renal tubules is a common outcome of ACE-I and ARB use, leading to intensified zincuria. Angiotensin II is a potent inducer of Na/H antiporter in the proximal tubule. Na/H antiporter facilitates renal Zn reabsorption. Thus, ACE-I and ARB lead to an angiotensin II blockade, causing diminished Na/H antiporter activity and intensified Zn loss in the urine [9]. However, our trial did not register an increase in zincuria after the use of ACE-I or ARB. We thus hypothesize that this mechanism depends on more complex factors, such as concomitant morbidity, pharmacological treatment, renal function, and duration of ACE-I/ARB treatment.

A unique result of our trial is the finding that erythrocyte Zn concentration decreases after Ca-antagonist pharmacotherapy in hypertensive patients. Zinc inhibits the activity of intracellular 1,4,5-triphosphoinositol-5-phosphatase (InsP3) [26]; this results in an accumulation of calcium inside the cell and an increase in arterial wall tension [27]. We thus hypothesize that decreased intracellular Zn content caused by Ca-antagonists may enhance hypotensive activity by decreasing arterial wall tension. This mechanism has been observed in rats [26,27], but further investigation is needed to confirm it in humans.

β-blockers lead to a decrease in renin secretion and, in consequence, to a drop in the concentration of angiotensin II [28]. We can thus theorize that this group of antihypertensive drugs may result in increased zincuria through the same mechanism involving ACE-I, ARB, and Na/H antiporter. However, studies have not yet been performed to confirm this molecular model [10], and our study has not noted Zn metabolism alterations in patients treated with β-blockers. 

In our trial, we saw a decrease in TG serum concentration following treatment with ACE-I. In the study of Suliburska et al. (2011) of 40 patients on hypotensive treatment, the concentration of serum TG was higher in hypertensive patients than in healthy controls. Moreover, hypertensive patients with higher TG serum concentrations (≥2.3 mmol/L) had higher serum concentrations of Fe and Zn and lower serum concentrations of Cu than hypertensive patients with lower TG serum levels (<2.3 mmol/L). In contrast with the current study, the patients in the trial of Suliburska et al. (2011) were encumbered by obesity and insulin resistance, and the drug groups used in the hypotensive therapy were not reported. In a rat model of hypertension, a lower level of serum TG was noted after metoprolol treatment than in the untreated hypertensive control group, with no alterations in serum TG concentrations in the group treated with the ACE-I perindopril [29]. However, the authors of [30] demonstrated that perindopril exerts no negative effect on serum lipids in hypertensive rats. Thus, our study not only confirms the observation of Swislocki in humans but also shows the favorable effect of ACE-I on TG blood concentrations.

In a rat model of arteriosclerosis [31], the Ca-antagonist amlodipine demonstrated an anti-inflammatory effect. The main mechanisms of the action of amlodipine are local inhibition of ACE, monocyte chemoattractant protein-1 (MCP-1), and rho-protein activity, coupled with a decrease in C-C chemokine receptor type 2 (CCR2) in circulating monocytes. In our study, we observed a decrease in TNF-α serum concentrations in the group of patients treated with Ca-antagonists, in which amlodipine predominated; this seems to be another anti-inflammatory mechanism of this drug group that has not previously been seen in human subjects. This observation confirms the results of our previous study on spontaneously hypertensive rats [32]. The increased blood concentrations of leukocytes (which did not, however, exceed the leukocytosis level) in patients treated with Ca-antagonists seem to be a compensatory reaction against the increased number of leukocytes with the invalid MCP-1/CCR2 pathway.

Excessive synthesis of reactive oxygen species (ROS) intensifies peroxynitrite formation, which disturbs endothelial nitric oxide synthase function and NO-mediated endothelial dilatation, resulting in hypertension [33,34]. Properly functioning mitochondria provide an efficient anti-oxidative system in the form of SOD and CAT activity, which prevents excessive ROS formation [35,36]. However, arterial hypertension is associated with a number of mitochondrial abnormalities, such as decreased mitochondrial mass and density. Arterial hypertension thus leads to impaired mitochondrial energy balance and dysfunction of the electron transport chain complex, which results in increased ROS production and increased oxidative stress [37,38]. The decrease in the serum concentration of SOD and CAT and the increase in the serum concentration of NO may be a marker of the decrease in ROS synthesis, diminished oxidative stress, and restored endothelial function after antihypertensive treatment and blood pressure reduction [36].

In arterial hypertension, SOD reduces BP and decreases vascular superoxide production. In AH, the copper chaperone for Cu/ZnSOD is upregulated, and Cu/ZnSOD activity increases [39]. Moreover, SOD production in AH is intensified by angiotensin II [40]. These mechanisms represent compensatory strategies, which preserve the resistance of arterioles and large vessel function, and prevent endothelial dysfunction [39,40]. In our trial, we observed a significant decrease in SOD serum levels after treatment with every group of drugs. Zn is an important cofactor of SOD [24]. We thus hypothesize that even a marginal decrease in serum Zn content in the course of antihypertensive treatment can lead to diminished SOD serum concentration. Furthermore, in the light of studies to date [39,40], the decrease in SOD serum level observed in our trial might have been a response to BP reduction [39] and decreased angiotensin II synthesis after therapy with ACE-I [40]. 

Catalase exerts a protective effect against AH development [41,42] and its decreased activity in the blood leads to a significant increase in BP [43]. On the other hand, the increased synthesis of catalase in the kidney precedes the development of AH in predisposed rats [44]. In our trial, we saw a significant decrease in serum CAT concentration after treatment with diuretics, Ca-antagonists, β-blockers, and ACE-I, which is indisputably a new finding in human hypertensive patients. The diminished CAT serum content seems to be a response to the decrease in BP caused by hypotensive treatment.

Hypertensive patients present a dysfunctional NO pathway, leading to decreased NO bioavailability [45,46]. In our study, we registered an increase in serum NO concentration after treatment with diuretics, β-blockers, ACE, and ARB. This confirms previous observations that antihypertensive drugs increase NO availability, activate NO signaling, attenuate NO inactivation, and reinforce the alternative nitrate–nitrite–NO pathway [46]. 

This study is the first anywhere to present the effect of three months of antihypertensive monotherapy on the mineral status of Zn, Fe, and Cu in serum, erythrocytes, urine, and hair in as many as 98 human subjects with newly diagnosed, not yet treated, primary AH, who were also not encumbered with other diseases. This is the strongest point of the study. Moreover, dietary control and the constant intake of nutrients, energy, Fe, Zn, and Cu with no differences between patients treated with different drug groups are also significant strong points of this study. The very homogenous group of subjects and the strict inclusion and exclusion criteria allowed us to draw a precise conclusion and to eliminate a large number of confounding factors. The results of our study include significant evidence that will be of use in revising guidelines on mineral supplementation during antihypertensive treatment.

The main limitation of the study is that the sex ratio in the study population was not close to 1:1, mainly due to the very strict inclusion and exclusion criteria. However, these criteria allowed us to avoid factors that might have prevented us from obtaining clear and unequivocal results. In addition, the number of patients receiving each drug from the various antihypertensive groups was diverse. However, patients received the studied drugs according to medical indications and contraindications, and in the line with the 2013 European Society of Hypertension guidelines [19]. A larger group of patients would have avoided this limitation.

## 5. Conclusions

We conclude that three-month antihypertensive monotherapy with diuretics, Ca-antagonists, and ACE-Is impairs zinc status in patients with newly diagnosed, previously untreated, and primary arterial hypertension. Modifications in zinc metabolism and antihypertensive pharmacotherapy are associated with a range of alterations in lipid metabolism and in oxidative and inflammatory states in this group of patients. These conclusions point to the urgent need for further large-scale studies on the recommendations regarding zinc supplementation during antihypertensive treatment.

## Figures and Tables

**Table 1:** Baseline characteristics and the amount of patients receiving particular antihypertensive monotherapy.

Parameter	Mean ± SD
*N*	98
Gender (F/M)	61/37
Age (year)	53.6 ± 13.7
BMI (kg/m^2^)	33.3 ± 8.7
WHR	0.9 ± 0.1
Monotherapy	*n*
Diuretic:	36
Indapamide	29
Torasemide	5
Spironolactone	2
Calcium antagonist	18
Amlodipine	13
Nitrendipine	5
β-blocker	18
Bisoprolol	7
Metoprolol	7
Nebivolol	4
Angiotensin-converting enzyme inhibitor	14
Perindopril	9
Captopril	3
Ramipril	2
Angiotensin II receptor antagonist	12
Losartan	8
Valsartan	2
Telmisartan	2

BMI—body mass index; F—female; M—male; *n*—number of subjects; SD—standard deviation; WHR—waist-hip ratio.

**Table 2:** Blood pressure, biochemical parameters, and complete blood count parameters in patients receiving particular antihypertensive monotherapy.

Parameter	Diuretics			Ca-Antagonists			β-Blockers			ACE-I			ARB		
	I Stage	II Stage	(Δ)	I Stage	II Stage	(Δ)	I Stage	II Stage	(Δ)	I Stage	II Stage	(Δ)	I Stage	II Stage	(Δ)
**SBP (mmHg)**	158.5 ± 16.1	136.7 ± 23.2 *	−0.14	161.9 ± 17.4	139.0 ± 13.3 *	−0.14	160.4 ± 17.9	141.6 ± 11.2 *	−0.12	162.1 ± 17.9	145.3 ± 13.8 *	−0.10	168.2 ± 21.1	146.2 ± 10.0 *	−0.13
**DBP (mmHg)**	91.3 ± 7.8	81.6 ± 9.2 *	−0.11	92.7 ± 8.5	86.4 ± 13.0 *	−0.07	90.1 ± 7.0	82.0 ± 5.5 *	−0.09	91.5 ± 8.1	80.6 ± 8.4 *	−0.12	95.4 ± 11.2	82.4 ± 10.8 *	−0.14
**TCH (mmol/L)**	5.9 ± 1.5	5.6 ± 1.1	−0.05	5.1 ± 0.9	4.9 ± 1.3	−0.04	4.8 ± 0.8	4.6 ± 1.0	−0.04	5.4 ± 0.8	4.9 ± 0.8	−0.09	5.7 ± 0.8	5.0 ± 1.2	−0.12
**LDL (mmol/L)**	3.2 ± 0.9	3.4 ± 1.1	0.06	2.8 ± 0.7	2.7 ± 1.0	−0.04	2.7 ± 0.6	2.7 ± 0.8	0.00	3.1 ± 0.6	2.9 ± 0.6	−0.06	3.5 ± 0.8	2.7 ± 1.0	−0.23
**HDL (mmol/L)**	1.5 ± 0.3	1.4 ± 0.3	−0.07	1.4 ± 0.3	1.6 ± 0.6	0.14	1.3 ± 0.3	1.2 ± 0.3	−0.08	1.3 ± 0.3	1.2 ± 0.4	−0.08	1.5 ± 0.3	1.5 ± 0.3	0.00
**TG (mmol/L)**	1.9 ± 0.9	1.9 ± 0.9	0.00	1.9 ± 0.7	2.2 ± 0.3	0.16	1.8 ± 0.6	1.6 ± 0.6	−0.11	2.5 ± 1.0	1.9 ± 0.6 *	−0.24	2.0 ± 0.6	1.5 ± 0.7	−0.25
**Glu (mmol/L)**	5.4 ± 1.0	5.6 ± 1.1	0.04	5.3 ± 0.4	5.4 ± 0.8	0.02	5.8 ± 2.0	5.4 ± 1.2	−0.07	5.6 ± 0.4	5.2 ± 0.3	−0.07	5.7 ± 0.9	5.9 ± 1.4	0.04
**Alb (g/L)**	42.6 ± 4.1	42.5 ± 4.5	0.00	42.3 ± 4.1	44.1 ± 6.1	0.04	41.9 ± 5.3	41.3 ± 5.7	−0.01	43.6 ± 3.3	43.4 ± 3.7	0.00	41.7 ± 4.8	42.3 ± 5.6	0.01
**CRP (mg/L)**	5.5 ± 2.4	5.0 ± 2.3	−0.09	4.5 ± 1.6	4.1 ± 2.3	−0.09	4.5 ± 2.7	4.8 ± 3.1	0.07	4.8 ± 2.0	4.5 ± 2.1	−0.06	5.1 ± 1.8	4.9 ± 2.1	−0.04
**TNF-α (pg/mL)**	4.4 ± 0.8	4.3 ± 0.8	−0.02	4.9 ± 0.9	3.8 ± 0.7 *	−0.22	4.6 ± 0.7	4.4 ± 0.8	−0.04	4.3 ± 0.8	4.4 ± 0.9	0.02	4.2 ± 0.8	3.9 ± 0.7	−0.07
**Ferr (µg/L)**	122.1 ± 102.1	127.2 ± 105.2	0.04	191.6 ± 120.6	139.9 ± 95.5	−0.27	115.1 ± 55.7	133.1 ± 117.8	0.16	128.4 ± 102.3	115.4 ± 94.1	−0.10	115.1 ± 105.2	186.8 ± 118.9	0.62
**Cer (g/L)**	0.4 ± 0.1	0.4 ± 0.2	0.00	0.4 ± 0.2	0.5 ± 0.3	0.25	0.4 ± 0.2	0.5 ± 0.3	0.25	0.4 ± 0.2	0.3 ± 0.1	−0.25	0.3 ± 0.05	0.3 ± 0.1	0.00
**TIBC (µmol/L)**	62.9 ± 11.5	58.7 ± 13.7	−0.07	53.3 ± 5.6	54.3 ± 5.5	0.02	66.2 ± 2.7	57.9 ± 11.4	−0.13	63.3 ± 12.1	53.4 ± 16.2	−0.16	65.4 ± 9.9	54.9 ± 8.4	−0.16
**NO (µmol/L)**	10.9 ± 3.4	14.9 ± 3.2 *	0.37	11.1 ± 2.0	13.7 ± 0.9	0.23	10.1 ± 2.4	14.2 ± 3.6 *	0.41	11.3 ± 1.0	16.0 ± 2.5 *	0.42	10.1 ± 1.9	16.5 ± 1.8 *	0.63
**CA (U/mL)**	4.4 ± 0.4	4.3 ± 0.5	−0.02	4.4 ± 0.5	4.5 ± 0.4	0.02	4.6 ± 0.2	4.5 ± 0.2	−0.02	4.5 ± 0.2	4.5 ± 0.2	0.00	4.8 ± 0.3	4.3 ± 0.5	−0.10
**CAT (U/gHg)**	76.3 ± 10.1	60.2 ± 4.9 *	−0.21	76.9 ± 8.1	63.6 ± 5.3*	−0.17	75.7 ± 11.0	64.1 ± 4.1 *	−0.15	75.5 ± 3.5	61.9 ± 5.1 *	−0.18	70.9 ± 9.3	64.3 ± 5.8	−0.09
**SOD (U/gHg)**	2301.9 ± 157.1	1908.6 ± 61.0 *	−0.17	2334.1 ± 159.1	1864.9 ± 54.2 *	−0.20	2279.6 ± 236.6	1699.9 ± 337.3 *	−0.25	2231.5 ± 250.3	1692.4 ± 342.5 *	−0.24	2170.1 ± 230.0	1865.1 ± 65.4 *	−0.14
**WBC (10^3^/µL)**	7.4 ± 2.2	7.2 ± 2.1	−0.03	6.8 ± 1.5	7.5 ± 1.7 *	0.10	7.4 ± 2.0	7.4 ± 3.3	0.00	8.6 ± 2.3	8.2 ± 2.4	−0.05	6.5 ± 1.1	8.2 ± 2.6	0.26
**RBC (10^6^/µL)**	4.7 ± 0.3	4.7 ± 0.4	0.00	4.6 ± 0.4	4.6 ± 0.4	0.00	4.8 ± 0.3	4.7 ± 0.3	−0.02	4.9 ± 0.3	4.8 ± 0.3	−0.02	4.8 ± 0.4	4.7 ± 0.3	−0.02
**Hgb (g/dL)**	9.0 ± 1.6	8.5 ± 0.5	−0.06	8.6 ± 0.7	8.2 ± 0.7	−0.05	9.4 ± 2.0	9.2 ± 1.9	−0.02	9.1 ± 1.5	8.6 ± 0.7	−0.05	9.3 ± 2.1	8.8 ± 0.6	−0.05
**HCT (%)**	41.4 ± 3.1	41.5 ± 2.7	0.00	40.2 ± 3.1	40.6 ± 3.4	0.01	43.0 ± 2.0	41.1 ± 2.8	−0.04	42.0 ± 2.9	42.5 ± 2.6	0.01	40.5 ± 4.4	41.6 ± 2.0	0.03

Data are presented as mean ± standard deviation (SD). ACE-I-angiotensin-converting enzyme inhibitors; Alb—albumin; ARB—angiotensin II receptor antagonists; CA—carbonic anhydrase; CAT—catalase; Cer—ceruloplasmin; CRP—C-reactive protein; DBP—diastolic blood pressure; Ferr—ferritin; Glu—glucose; HCT—hematocrit; HDL—high-density lipoprotein; Hgb—hemoglobin; LDL—low-density lipoprotein cholesterol; NO—nitric oxide; RBC—red blood cells; SBP—systolic blood pressure; SOD—superoxide dismutase; TCH—total cholesterol; TG—triglycerides; TIBC—total iron binding capacity; TNF-α—tumor necrosis factor α; WBC—white blood cells; (Δ)—relative change [(Δ) = (II stage value − I stage value)/I stage value]; *—significant difference (*p* < 0.05) compared to stage I. The *n*-value of each analyzed study group is presented in Table 1.

**Table 3:** Iron (Fe), zinc (Zn), and copper (Cu) content in patients receiving particular antihypertensive monotherapy.

Parameter	Diuretics			Ca-Antagonists			Β-blockers			ACE-I			ARB		
	I Stage	II Stage	(Δ)	I Stage	II Stage	(Δ)	I Stage	II Stage	(Δ)	I Stage	II Stage	(Δ)	I Stage	II Stage	(Δ)
**Serum (µmol/L)**															
**Fe**	16.5 ± 7.0	17.7 ± 6.2	0.07	19.2 ± 6.8	18.8 ± 8.3	−0.02	16.5 ± 6.1	16.7 ± 6.6	0.01	18.0 ± 8.1	17.9 ± 7.4	−0.01	17.9 ± 5.0	18.5 ± 5.6	0.03
**Zn**	10.1 ± 1.5	9.1 ± 1.2 *	−0.10	9.8 ± 1.8	9.5 ± 1.5	−0.03	9.9 ± 1.7	9.8 ± 1.5	−0.01	10.2 ± 1.8	9.4 ± 1.5 *	−0.08	11.2 ± 1.8	9.9 ± 1.5	−0.12
**Cu**	15.3 ± 2.0	15.5 ± 2.1	0.01	16.4 ± 3.1	16.3 ± 2.1	−0.01	15.7 ± 3.0	15.2 ± 2.9	−0.03	15.3 ± 2.1	17.2 ± 2.3	0.12	14.7 ± 1.6	15.0 ± 1.4	0.02
**Erythrocytes (µmol/g Hgb)**															
**Fe**	48.9 ± 8.1	49.9 ± 8.7	0.02	51.2 ± 7.4	53.4 ± 8.3	0.04	48.8 ± 7.3	53.2 ± 9.5	0.09	49.8 ± 9.8	53.6 ± 12.0	0.08	49.6 ± 9.4	53.6 ± 11.2	0.08
**Zn**	0.5 ± 0.1	0.4 ± 0.1 *	−0.20	0.5 ± 0.1	0.4 ± 0.1 *	−0.20	0.5 ± 0.1	0.5 ± 0.1	0.00	0.4 ± 0.1	0.4 ± 0.1	0.00	0.4 ± 0.1	0.4 ± 0.1	0.00
**Cu**	44.0 ± 10.2	46.1 ± 10.6	0.05	40.3 ± 10.4	44.5 ± 11.0	0.10	41.6 ± 10.6	42.7 ± 9.7	0.03	41.9 ± 10.9	43.0 ± 10.2	0.03	44.6 ± 10.5	43.2 ± 9.6	−0.03
**Urine (µmol/24 h)**															
**Fe**	2.1 ± 1.2	2.3 ± 1.2	0.10	2.2 ± 0.9	2.1 ± 0.7	−0.05	2.2 ± 0.8	3.1 ± 1.9	0.41	1.2 ± 0.2	1.6 ± 0.4	0.33	2.2 ± 0.9	2.0 ± 0.9	−0.09
**Zn**	4.8 ± 2.0	7.2 ± 1.8 *	0.50	6.0 ± 2.0	7.3 ± 2.1	0.22	5.2 ± 1.4	5.5 ± 1.2	0.06	5.6 ± 1.9	5.7 ± 2.0	0.02	6.4 ± 2.6	6.1 ± 1.9	−0.05
**Cu**	0.7 ± 0.2	0.8 ± 0.2	0.14	0.8 ± 0.2	0.8 ± 0.2	0.00	0.7 ± 0.3	0.8 ± 0.3	0.14	0.8 ± 0.4	0.6 ± 0.1	−0.25	1.0 ± 0.3	1.1 ± 0.3	0.10
**Hair (µg/g)**															
**Fe**	16.0 ± 4.1	17.5 ± 4.0	0.09	14.4 ± 1.8	13.5 ± 1.7	−0.06	13.9 ± 4.3	14.3 ± 4.9	0.03	17.6 ± 3.2	16.2 ± 3.3	−0.08	9.8 ± 5.6	8.9 ± 5.4	−0.09
**Zn**	134.9 ± 25.9	131.9 ± 24.8	−0.02	123.2 ± 19.9	119.2 ± 19.1	−0.03	135.1 ± 21.1	128.2 ± 20.1	−0.05	109.8 ± 30.2	104.2 ± 28.9	−0.05	122.2 ± 20.0	117.1 ± 19.3	−0.04
**Cu**	14.9 ± 6.2	15.6 ± 6.4	0.05	14.3 ± 5.0	13.0 ± 4.1	−0.09	20.1 ± 12.1	21.6 ± 13.1	0.07	15.7 ± 9.6	16.6 ± 10.5	0.06	20.7 ± 5.5	21.5 ± 6.1	0.04

Data are presented as mean ± standard deviation (SD). ACE-I—angiotensin-converting enzyme inhibitors; ARB—angiotensin II receptor antagonists; Hgb—hemoglobin; (Δ)—relative change [(Δ) = (II stage value − I stage value)/I stage value]; *—significant difference (*p* < 0.05) compared to stage I. The *n*-value of each analyzed study group is presented in Table 1.
